# Aneurysmal formation of periventricular anastomosis is associated with collateral development of Moyamoya disease and its rupture portends poor prognosis: detailed analysis by multivariate statistical and machine learning approaches

**DOI:** 10.1007/s10143-024-03097-2

**Published:** 2024-11-19

**Authors:** Daisuke Sato, Satoru Miyawaki, Seiei Torazawa, Hideaki Imai, Hiroki Hongo, Satoshi Kiyofuji, Satoshi Koizumi, Nobuhito Saito

**Affiliations:** 1 Department of Neurosurgery, Faculty of Medicine, The University of Tokyo, 7-3-1 Hongo, Bunkyo-ku, Tokyo, Japan; 2 Department of Neurosurgery, JCHO, Tokyo Shinjuku Medical Center, 5-1 Tsukudo-cho, Shinjuku-ku, Tokyo, Japan

**Keywords:** Moyamoya disease, Periventricular anastomosis, Aneurysm, Choroidal anastomosis, Machine learning

## Abstract

Periventricular anastomosis (PA) is the characteristic collateral network in Moyamoya disease (MMD). However, PA aneurysms are rare, resulting in limited knowledge of their clinical significance. We aimed to elucidate the associated factors and clinical outcomes of PA aneurysms. We reviewed MMD patients who underwent digital subtraction angiography in our institution between December 2001 and March 2023. Genetic analysis was conducted in several cases, and PA aneurysm-positive patients were identified. PA score was defined as the grades of development of periventricular anastomosis. Multivariate analysis and machine learning approaches were used to investigate the significance of the disease and factors associated with PA aneurysm positivity. A total of 301 hemispheres (171 patients) were included. PA aneurysm occurred in 8 hemispheres of MMD (2.7%). PA aneurysm was associated with higher initial modified Rankin scale (mRS) scores (OR, 2.61; 95% CI, 1.45–4.70) and higher PA scores (OR, 1.60; 95% CI, 1.06–2.40). This predisposition was corroborated by gradient boosting and random forest algorithms. Further analysis revealed that PA aneurysm was a risk factor for future hemorrhagic stroke events (HR, 8.29; 95% CI, 1.44–47.7). Among patients in the hemorrhagic-onset group (33 cases), PA aneurysm was a risk factor for worse outcomes (*P* = 0.008). Principal component analysis also revealed distinct characteristics of hemorrhagic onset aneurysms compared to other MMD cases. Higher PA scores were associated with the presence of aneurysm. PA aneurysm suggests a higher risk of future hemorrhagic strokes, and its rupture portends a worse prognosis.

**Supplementary Information:**

The online version contains supplementary material available at 10.1007/s10143-024-03097-2.

## Introduction

Moyamoya disease (MMD) is a progressive steno-occlusive disease affecting the terminus of internal carotid arteries and proximal portions of the anterior and middle cerebral arteries [1]. The *ring finger protein 213* (*RNF213*) gene is a well-known factor in MMD development, and its variant (*RNF213* p.Arg4810Lys, c.14429G > A　rs112735431) has been reported in up to 80% of patients with MMD [2, 3]. One hallmark of the disease is the compensatory development of fine collateral networks to bypass the stenotic lesion [4]. Periventricular anastomosis (PA) is a characteristic collateral network of MMD that is known for its delicate connection between the perforating periventricular and medullary arteries [5]. PA can be classified into three subtypes - lenticulostriate, thalamic, and choroidal [5–7], of which choroidal anastomosis has been demonstrated as a strong predictor for hemorrhagic strokes [8, 9].

Aneurysms arising from the PA are rarely encountered [10]. Specifically, distal choroidal artery (ChA) [4], lenticulostriate artery (LSA) [11], and thalamic artery (THA) aneurysms [12, 13] are possible pathologies in this area. These aneurysms can be located intraventricularly [10] or periventricularly [13] and pose a significant risk of hemorrhage and consequent devastating outcomes [14–16].

PA aneurysms are generally caused by hemodynamic stress and fragile vascular changes in the intracranial vessels [17]. The incidence ranges from 2.3 to 24.7% [18, 19], and association with hemorrhagic stroke has been occasionally described [17, 18]. However, the risk factors, genetic analysis, difference in the clinical course between ruptured and unruptured aneurysm, and long-term outcomes are not fully investigated.

Herein, we described the pathophysiology of PA aneurysms in patients with MMD and aimed to elucidate the epidemiology, clinical features, and significance of this lesion by reviewing our single-center experience.

## Materials and methods

### Patient selection

This study was approved by the Ethics Committee of the Faculty of Medicine, University of Tokyo (approval number 2231, G10026), and written informed consent was obtained from all participants. We included our experience with 171 consecutive patients with MMD (301 hemispheres) who underwent digital subtraction angiography at our institution between December 2001 and March 2023. MMD diagnosis was confirmed using the diagnostic criteria published by the Research Committee on Moyamoya Disease (Spontaneous Occlusion of the Circle of Willis) of the Ministry of Health and Welfare of Japan [20]. Patients with hemorrhagic and ischemic presentations were included, as well as asymptomatic cases. Quasi-moyamoya disease was excluded from our cohort.

### Clinical-radiological evaluation

Medical records and radiological imaging of each patient were scrutinized by three independent authors (D.S., S.T., and S.M.). The reviewed data included age, symptomatology, history of presentation, past medical history, affected side, computed tomography (CT) scans, magnetic resonance (MR) images, digital subtraction angiography (DSA) studies, date of operation, methods of revascularization, cerebrovascular events during the follow-up period, and the last follow-up date. Modified Rankin scale (mRS) scores were obtained from medical records, follow-up visits, and telephone interviews.

DSA studies were analyzed in detail, and each case was classified based on the Suzuki grading system [21]. PA aneurysms were classified into three categories (ChA, LSA, and THA aneurysms). The degree of PA development was evaluated based on the angiographic criteria described in previous reports [6, 7]. Choroidal artery collaterals were categorized into the anterior choroidal artery (AChA) and posterior choroidal artery (PChA) collaterals for further analysis. ChA collateral grading was defined as the combined scores of AChA and PChA collaterals, taking the higher value for each. The degree of collateral vessel development was graded on a three-point scale (0–2), with Grade 0 indicating dilatation and extension, Grade 1 signifying intermediate findings, and Grade 2 indicating remarkable dilatation and extension. The grading of all collateral anastomoses was evaluated based on the definition described by Ryu et al. [6]. The PA scoring system is illustrated in Supplement 1. The rationale for the grading used in this study is that it allows for a detailed evaluation of the development and provides a quantitative description. Furthermore, the validity is already testified by a large cohort. PA score was calculated as the sum of the individual grades of each collateral pathway. Notably, a PA score of 1 or higher was considered “PA positive”, whereas a PS score of more than 2 was considered “higher PA.” Posterior cerebral artery (PCA) involvement was defined as an occlusion or stenosis of more than 50% in the P1 to P3 segments. All angiographic images were reviewed by the two independent authors (D.S. and S.T.). When there was a disagreement among the raters’ assessments, the evaluations were determined through discussion and validated by another author (S.M.). The investigators were blinded to the patients’ clinical information during the evaluation.

### Genetic evaluation

DNA analysis was performed for certain patients. Genomic DNA was extracted from peripheral white blood cells, and direct Sanger sequencing was conducted to analyze the *RNF*213 p.Arg4810Lys gene using NM_001256071 as the reference sequence. The genetic evaluation method in this paper has been reported in our previous study [22].

### Statistical analyses

Comparisons between groups were performed using the Mann-Whitney U test and Kruskal-Wallis test for continuous variables and Fisher’s exact test for categorical variables as appropriate.

To identify the factors associated with PA aneurysm occurrence, multivariate analysis and machine learning algorithms were applied. For the multivariate analysis, 14 variables (sex, age, childhood onset, initial mRS score, Suzuki grade, PA score, PCA involvement, hypertension, dyslipidemia, diabetes mellitus, ischemic heart disease, thyroid disease, hemorrhagic onset, and ischemic onset) were examined. Involved factors were determined with stepwise backward selection using p-values, and the area under the curve (AUC) of the receiver operating characteristic (ROC) curve was calculated to distinguish cases with PA aneurysms. For machine learning algorithms, 16 variables (sex, age, childhood onset, initial mRS score, Suzuki grade, PA score, PCA involvement, hypertension, dyslipidemia, diabetes mellitus, ischemic heart disease, thyroid disease, hemorrhagic onset, ischemic onset, *RNF*213 GA genotype, and *RNF*213 GG genotype) were examined. For cases that did not undergo genotyping, those were classified as *RNF213* GA genotype negative and *RNF213* GG genotype negative in the machine learning approaches. We randomly split the data into the training and test datasets in a 3:1 ratio. Based on the training dataset, we developed four machine-learning models: random forest, SVM, gradient boosting, and multi-layer perceptron models. The AUC of the ROC curve was calculated for each model to assess performance, and five-fold cross-validation and grid search were utilized for feature selection and hyperparameters optimization. Based on the AUC of each model, we selected the optimal model, which was validated using the test dataset. Furthermore, we visualized variable importance in the selected model.

The cumulative incidence of all strokes and hemorrhagic strokes was calculated using the Kaplan-Meier method. We excluded data at the point of undergoing surgery. The multivariate Cox proportional hazards regression model was used to examine factors associated with ischemic stroke events and hemorrhagic stroke during the follow-up period. Focusing on hemorrhagic-onset cases, multivariate linear regression analysis was additionally performed to explore the association between select clinical factors and final mRS scores. Eleven variables (sex, age, initial mRS score, Suzuki grade, PA score, PA aneurysm, PCA involvement, hypertension, dyslipidemia, diabetes, and ischemic heart disease) were examined. To avoid multicollinearity, the Akaike information criterion (AIC) and the algorithm stepAIC were conducted to select the most accurate linear regression model. The threshold for significance in all statistical analyses was set at *P* < 0.05.

Principal component analysis was also performed to analyze and reduce the dimensionality of.

the dataset. The analysis was conducted with 17 variables (sex, age, childhood-onset, initial mRS score, Suzuki grade, PA score, PA aneurysm, PCA involvement, hypertension, dyslipidemia, ischemic heart disease, thyroid disease, last mRS score, hemorrhagic onset, ischemic onset, *RNF213* GA genotype, and *RNF213* GG genotype). Specifically, clusters of cases were identified based on individual resemblance and were evaluated to determine distinctions between MMD patients with and without aneurysms.

Statistical analyses were conducted using EZR (Saitama Medical Center, Jichi Medical University, Saitama, Japan), a graphical user interface for R software (The R Foundation for Statistical Computing, Vienna, Austria) [23], whereas machine learning approaches were conducted using Python (Python Software Foundation, Delaware, the U.S.).

## Results

### Characteristics of the study cohort

Baseline characteristics of the study population are described in Table 1. A total of 171 patients (301 hemispheres) were included in the study, predominantly females (218/301, 72.4%) with an age range of 5–81 years. Among the 164 cases (54.5%) who underwent *RNF213* p.Arg4810Lys analysis, 53 cases (32.3%) carried the GG phenotype, while 111 cases (67.7%) carried the GA genotype. The follow-up period was 46.6 ± 53.2 months (range, 0–252 months), and 145 hemispheres (48.2%) underwent revascularization surgery during this time.

**Table 1:** Baseline characteristics of the study population

Parameters	All	MMD hemispheres with PA aneurysms	MMD hemispheres without PA aneurysms	*p*-value
Number of hemispheres	301	8	293	
Average age at presentation (range)	39 (5–81)	42 (7–81)	39(5–81)	0.82
Female	218 (72.4%)	7 (87.5%)	211 (72.0%)	0.45
Presentation				
Hemorrhagic presentation	33 (11.0%)	4 (50.0%)	29 (9.9%)	**0.006**
Ischemic presentation	157 (52.2%)	3 (37.5%)	154 (52.6%)	0.49
Asymptomatic	111 (36.9%)	1 (12.5%)	110 (37.5%)	0.27
Childhood onset	46 (15.3%)	2 (25.0%)	44 (15.0%)	0.35
*RNF213* p.Arg4810Lys variant				
Genotype Examined	164	5	159	
GG genotype	53 (32.3%)	3 (60.0%)	50 (31.4%)	0.33
GA genotype	111 (67.7%)	2 (40.0%)	109 (68.6%)	
**PA**				
** PA score, median (IQR)**	**1 (0–3)**	**3 (3–4)**	**1 (0–3)**	**0.003**
** PA positive**	**186 (61.8%)**	**8 (100%)**	**178 (60.8%)**	**0.026**
** higher PA (PA > = 3)**	**86 (28.6%)**	**7 (87.5%)**	**79 (27.0%)**	**< 0.001**
Suzuki grade, mean ± SD	2.79 ± 1.04	3.13 ± 0.64	2.78 ± 1.04	0.35
PCA involvement	38 (12.6%)	2 (25.0%)	36 (12.3%)	0.27
Comorbidities				
Hypertension	93 (30.9%)	4 (50%)	89 (30.4%)	0.26
Diabetes	22 (7.3%)	0 (0%)	22 (7.5%)	1
Dyslipidemia	28 (9.3%)	0 (0%)	28 (9.6%)	1
Thyroid disease	12 (4.0%)	1 (12.5%)	11 (3.8%)	0.28
Ischemic heart disease	4 (1.3%)	0 (0%)	4 (1.4%)	1
**Initial mRS score, median (IQR)**	**2 (2–2)**	**2.5 (2–4.25)**	**2 (2–2)**	**0.002**

IQR, interquartile range; MMD, moyamoya disease; mRS, modified Rankin scale; PA, periventricular anastomosis

Hemorrhagic, ischemic, and asymptomatic presentations were found in 33, 157, and 111 cases, respectively. Among them, eight PA aneurysms were discovered in seven patients, indicating a prevalence of 2.7%. Specifically, PA aneurysms were discovered in four cases (12.2%) of hemorrhagic presentation, three cases (1.9%) of ischemic presentation, and one case (0.9%) of asymptomatic presentation, all of which were statistically significant (*P* = 0.002). Further p-value adjustments using Bonferroni’s method revealed significant differences between hemorrhagic and ischemic presentation (*P* = 0.015) and between hemorrhagic and asymptomatic presentation (*P* = 0.006).

Comparisons between MMD patients with and without PA aneurysms are also shown in Table 1. Although no significant differences were observed in age, sex, *RNF 213* p.Arg4810Lys variant, Suzuki grade, PCA involvement, and comorbidities (hypertension, diabetes, dyslipidemia, thyroid disease, and ischemic heart disease), differences in PA score, PA positivity, and higher PA were statistically significant (*P* = 0.003, *P* = 0.026, and *P* < 0.001, respectively). Similarly, initial mRS scores were statistically different between the two groups (*P* = 0.002).

### Reliability of radiological evaluation

Interrater (D.S. and S.T.) reliability for the presence of each subtype of periventricular anastomosis was almost complete (lenticulostriate, κ = 0.860; choroidal, κ = 0.927; thalamic, κ = 0.881). When there was a disagreement among the raters’ assessments, the evaluations were determined through discussion and validated by another author (S.M.).

### Location and presentation of PA aneurysms

**Fig. 1:**
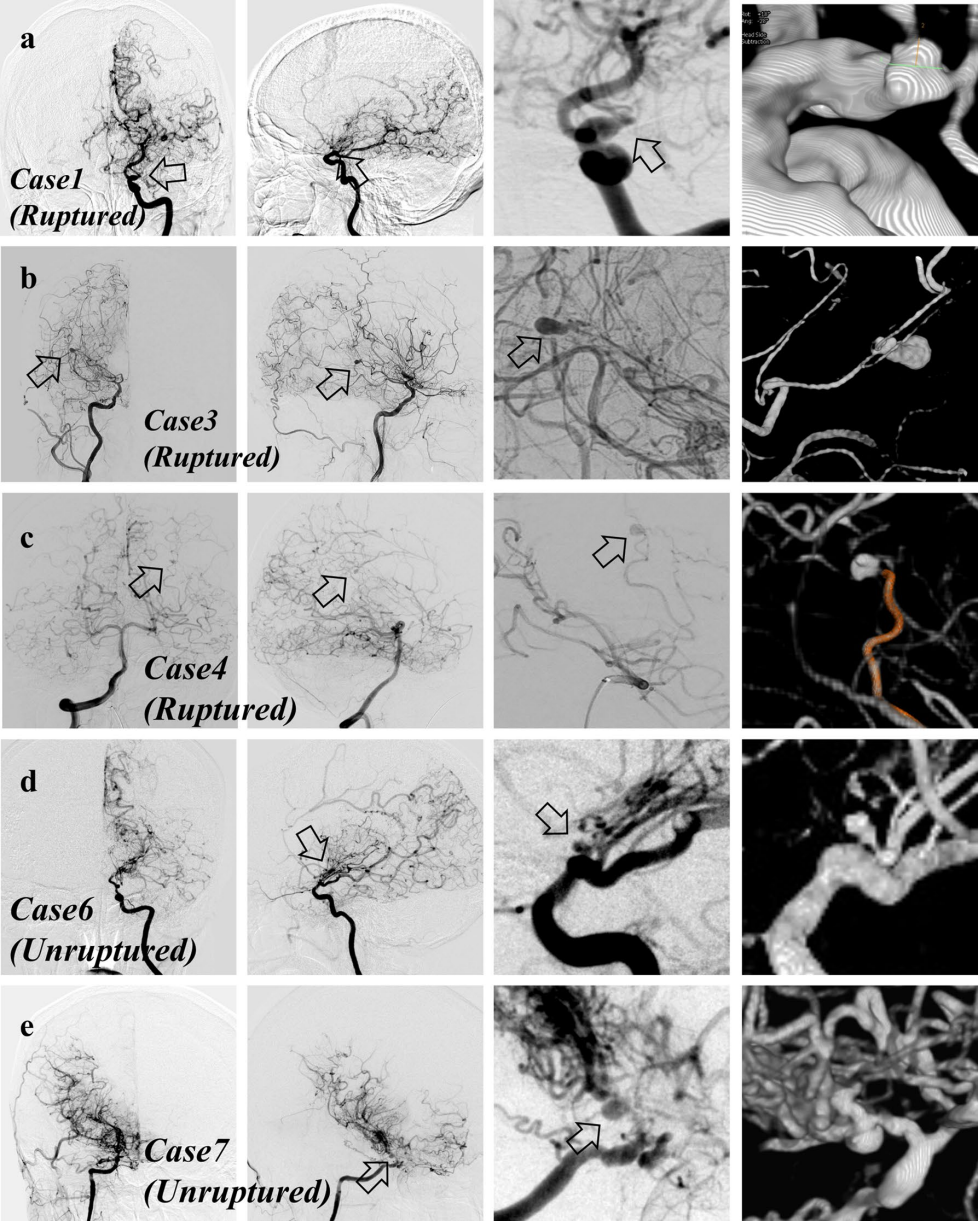
Angiograms of periventricular anastomosis (PA) aneurysm-positive cases are demonstrated. Cases 1, 3, 4, 6, and 7 are labeled as **a**, **b**, **c**, **d**, and **e**, respectively. The far left corresponds to the anteroposterior view, and the center-left shows the lateral view. The center-right shows the magnified view, and the far right shows the rotational three-dimensional imaging. The arrow indicates the aneurysm

PA aneurysms were found in eight hemispheres. Several aneurysms are shown in Fig. 1, and the remaining cases are shown in Supplement 2. Patient geographics, aneurysmal locations, and clinical presentations are summarized in Table 2. Additional images of the hemorrhagic-onset group are depicted in Supplement 3.

**Table 2:** Clinical summary of the seven patients with periventricular anastomosis aneurysms

Case Number	Age/sex	Presentation	Aneurysm status	Location	Laterality	Initial mRS score	PA score	RNF213 *p*.Arg4810Lys variant	Initial surgery	Interval of presentation and surgery	Stroke during the follow-up period	Aneurysmal re-rupture	Stroke-free period	Surgery after recurrent stroke	FU period	Last mRS score
1	81y/F	SAH, ICH	Ruptured	AchA	L	4	2	GG	None	-	Yes (hemorrhage)	No	39 months	Bilateral VD and following VPS	43 months	5
2	38y/F	IVH	Ruptured	LPChA	R	5	6	GA	STA-MCA bypass + EMS	3 months	Yes (hemorrhage)	No	39 months	None	142 months	5
3	69y/F	IVH	Ruptured	AchA	R	3	3	-	None	-	Yes (hemorrhage)	Yes	5 days	Endoscopic hematoma evacuation, TAE, and following VPS	2 months	5
4	54/M	IVH	Ruptured	LPChA	L	5	3	GG	TAE	15 days	No	-	4 months	-	4 months	5
5	7y/F	TIA	Unruptured	THA	R	2	4	-	EDAS + EMS	7 days	No	-	20 days	-	20 days	2
			Unruptured	THA	L	2	5	-	None	-	No	-	20 days	-		
6	39y/F	TIA	Unruptured	AchA	L	2	2	GG	STA-MCA bypass + EMS	29 days	No	-	13 months	-	13 months	0
7	40y/F	TIA	Unruptured	AchA	L	2	3	GA	STA-MCA bypass + EMS	2 months	No	-	127 months	-	127 months	0

AchA, anterior choroidal artery; EDAS, encephalo-duro-arterio-synangiosis; EMS, encephalo-myo-synangiosis; FU, follow-up; ICH, intracerebral hemorrhage; IVH, intraventricular hemorrhage; L, left; LPChA, lateral posterior choroidal artery; MCA, middle cerebral artery; mRS, modified Rankin scale; PA periventricular anastomosis; R, right; STA, superficial temporal artery; TAE, trans-arterial embolization; THA, thalamic artery; SAH, subarachnoid hemorrhage; VD, ventricular drainage; VPS, ventriculo-peritoneal shunt

In our cohort, four cases with ruptured aneurysms presented with hemorrhagic stroke, while the remaining four cases were unruptured and presented with ischemic stroke. Three cases presented with intraventricular hemorrhage, and a single case presented as subarachnoid hemorrhage and intraparenchymal hemorrhage. Regarding location, six cases were diagnosed with ChA aneurysms, two with THA aneurysms, and no diagnosis with LSA aneurysms was made. For genetic evaluation, five cases underwent *RNF213* analysis. Two out of three ruptured aneurysm cases carried the GG genotype, whereas one out of two unruptured aneurysm cases carried the GG genotype.

Three out of the four ruptured aneurysm cases experienced recurrent hemorrhagic stroke and were bedridden at the last follow-up. In particular, one case experienced a re-rupture of the aneurysm 5 days after the initial hemorrhage (*Case 3*). Despite undergoing endoscopic hematoma evacuation, transarterial embolization, and ventriculoperitoneal shunt, the patient sustained deep white matter infarction due to the damaged vessels supplying the PA, leaving her bedridden until the day of her last follow-up. Two other cases (*Case 1* and *Case* 2) suffered from intracerebral hemorrhages approximately 3 years after their initial stroke. Notably, both cases revealed hemorrhages that were not adjacent to the aneurysm. Both patients were also bedridden at the latest follow-up. For the remaining case that did not experience a recurrent hemorrhagic stroke (*Case 4*), the patient underwent transarterial embolization immediately after presentation. Although the aneurysm was completely obliterated, the patient remained bedridden due to initial brain damage.

In contrast, none of the unruptured aneurysm cases experienced hemorrhagic stroke or rupture. All four cases were living independently at the last follow-up.

### Factors associated with PA aneurysm occurrence

Clinical factors associated with PA aneurysm occurrence were examined as follows. First, univariate analysis was performed, revealing higher PA scores (*P* = 0.003) and higher initial mRS scores (*P* = 0.002) among PA aneurysm-positive cases.

**Fig. 2:**
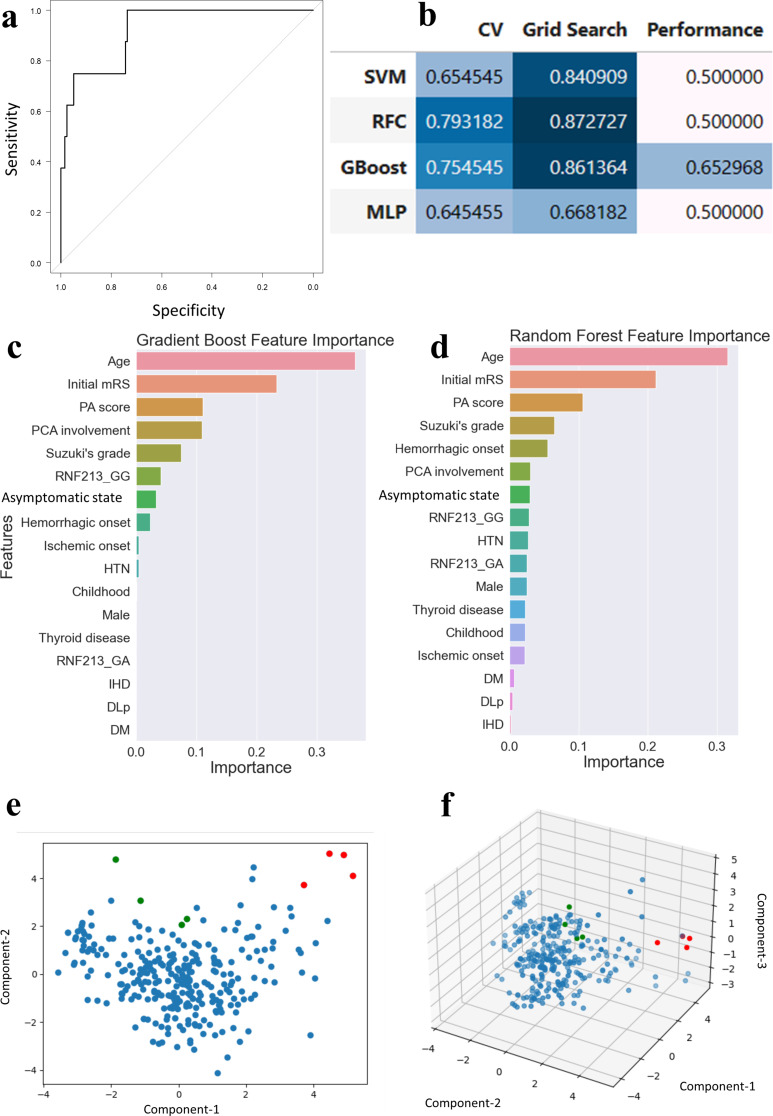
Multivariate analysis revealed factors associated with periventricular anastomosis (PA) aneurysm and depicted the receiver operating characteristic (ROC) curve (**a**). The area under the curve (AUC) was 0.90 (95% confidence interval, 0.83–0.97). Machine learning approaches were used to examine factors associated with the aneurysm, and performance was calculated (**b**). The AUC of five-fold cross-validation (CV), grid search for hyperparameters tuning, and performance on test data were calculated, respectively. Gradient boosting achieved the highest performance. To visualize the influence of variables on aneurysm occurrence, the feature importance of each variable was calculated according to the gradient boosting (**c**) and random forest model (**d**). Both models demonstrate age, initial modified Rankin Scale (mRS) score, and PA score as significant associations in descending order. Principal component analysis was performed with 17 variables. First, the dimension was reduced to two (**e**). Ruptured and unruptured PA aneurysm cases are displayed as red and green dots, respectively, whereas other cases are displayed as blue dots. Ruptured PA aneurysm cases were distinct from other cases, while unruptured PA aneurysm cases were relatively indistinguishable. Thereafter, the dimension was reduced to three (**f**), which revealed the same tendency for distinction among ruptured PA aneurysm cases

AUC, area under the curve; CV, cross-validation; DLp, dyslipidemia; DM, diabetes mellitus; GBoost, gradient boosting; HTN, hypertension; IHD, ischemic heart disease; MLP, multi-layer perceptron; mRS, modified Rankin scale; PA, periventricular anastomosis; PCA, posterior cerebral artery; RFC, random forest classifier; SVM, support vector machine.

Second, multivariate analysis showed that PA aneurysm was associated with higher initial mRS scores (odds ratio, 2.61; 95% confidence interval [CI], 1.45–4.70) and higher PA scores (odds ratio, 1.60; 95% CI, 1.06–2.40). The ROC curve is depicted in Fig. 2a, demonstrating an AUC of 0.92 (95% CI, 0.84–1.00).

Lastly, machine learning approaches were used. The performance of each model is presented in Fig. 2b. Random forest achieved the highest performance, followed by gradient boosting, showing an AUC of 0.65 on the dependent test dataset. To visualize the influence of variables to aneurysm occurrence, variable importance was plotted according to the gradient boosting (Fig. 2c) and random forest (Fig. 2d) models. Both models revealed that age, initial mRS score, and PA score were associated with PA aneurysm occurrence in order of descending importance.

### Clinical outcomes of PA aneurysms

#### Future stroke

During the study period, 20 strokes were observed in our cohort, including 11 hemorrhagic strokes and nine ischemic strokes. It should be noted that this rate excludes the influence of surgery, since we excluded data collected at the point of undergoing surgery. For ischemic stroke, the 1-year, 3-year, 5-year, and 10-year cumulative rates were 0%, 2.7%, 6.6%, and 11.3%, respectively (Supplement 4a). Hemorrhagic strokes followed a similar trend, with 1-year, 3-year, 5-year, and 10-year cumulative rates of 1.5%, 4.9%, 8.3%, and 13.4%, respectively (Supplement 4b). Focusing on the hemorrhagic-onset group (33 cases), five patients who presented with hemorrhagic stroke experienced a second hemorrhagic stroke during the follow-up period. The 1-year, 3-year, 5-year, and 10-year cumulative rates for this group were 3.2%, 10.1%, 46.1% and 46.1%, respectively (Supplement 4c). PA aneurysm-positive groups and PA aneurysm-negative groups were compared for the risk of future hemorrhagic strokes (Supplement 4d). A log-rank test was executed, showing that PA aneurysm-positive groups were prone to experience future hemorrhagic stroke events (*p* < 0.001).

Risk factors for future hemorrhagic and ischemic stroke events were analyzed. Although no significant risk factors for ischemic stroke events were evident after univariate analysis, several risk factors for hemorrhagic stroke events showed significant associations (Table 3). The stepAIC algorithm was then utilized to narrow down the five risk factors to three significant risk factors: PA score (hazard ratio, 1.40; 95% CI, 1.03–1.92), PA aneurysm (hazard ratio, 8.29; 95% CI, 1.44–47.7), and initial mRS score (hazard ratio, 1.66; 95% CI, 1.05–2.62).

**Table 3:** Univariate and multivariate analysis results to determine the risk factors for hemorrhagic stroke events

Variables	Univariate		Multivariate	
	p-value	Hazard Ratio (95% Confidence Interval)	p-value	Hazard Ratio (95% Confidence Interval)
Age	0.33	1.02 (0.98–1.07)	-	-
Sex	0.83	0.87 (0.23–3.28)	-	-
**Hemorrhagic onset**	**< 0.001**	**8.42 (2.42**–**29.3)**	-	-
Ischemic onset	0.28	0.43 (0.09–1.99)	-	-
PCA involvement	0.63	1.47 (0.32–6.83)	-	-
**Suzuki grade**	**0.006**	**2.13 (1.24**–**3.64)**	-	-
Hypertension	0.40	1.67 (0.51–5.50)	-	-
Dyslipidemia	0.55	0.54 (0.07–4.20)	-	-
Diabetes	n/a	n/a	-	-
Ischemic heart disease	n/a	n/a	-	-
Thyroid disease	n/a	n/a	-	-
**PA score**	**0.012**	**1.40 (1.08**–**1.81)**	**0.034**	**1.40 (1.03**–**1.92)**
**PA aneurysm**	**< 0.001**	**23.4 (4.82**–**113.6)**	**0.018**	**8.29 (1.44**–**47.7)**
**Initial mRS score**	**< 0.001**	**2.16 (1.37**–**3.40)**	**0.032**	**1.66 (1.05**–**2.62)**

mRS, modified Rankin scale; n/a, not applicable; PA, periventricular anastomosis; PCA, posterior cerebral artery

### Functional outcomes

The mRS scores at the last follow-up were evaluated to assess functional outcomes. The median mRS scores were 2.0, 0.0, and 0.0 for hemorrhagic, ischemic, and asymptomatic presentations, respectively. Differences between groups were statistically significant (*P* < 0.001) on the Kruskal-Wallis test. Pairwise comparisons using the Mann-Whitney U-test and p-value adjustment using Bonferroni’s method revealed significant differences between hemorrhagic and ischemic presentations (*P* < 0.001) and between hemorrhagic and asymptomatic presentations (*P* = 0.001).

Factors affecting the last mRS scores of the hemorrhagic-onset group were further evaluated. Multivariate analysis was performed to explore the association between several clinical factors and last mRS scores. The Akaike Information Criterion (AIC) and the algorithm stepAIC were conducted, as demonstrated in Table 4. Among them, the initial mRS score, age, PA score, and PA aneurysm affected the last mRS score; the model was predicted as follows: last mRS = -2.86 + 0.81 x (initial mRS) + 0.030 x (age) + 0.37 x (PA score) + 1.49 x (PA aneurysm). This model, with an adjusted R-squared of 0.83, showed that PA aneurysm strongly influenced the last mRS score (coefficient: 1.49).

**Table 4:** Multivariate analysis results to determine the risk factors for poor prognosis (higher last modified Rankin scale)

Variables	Estimate	Standard Error	t-value	*p*-value
(Intercept)	-2.86	0.72	-3.98	< 0.001
Initial mRS score	0.81	0.14	5.67	< 0.001
Age	0.030	0.012	2.56	0.016
PA score	0.37	0.089	4.11	< 0.001
**PA aneurysm**	**1.49**	0.53	2.84	**0.008**

Prediction Model: Last mRS = -2.86 + 0.81 x (Initial mRS) + 0.030 x (Age) + 0.37 x (PA score) + 1.49 x (PA aneurysm)

Multiple R-squared: 0.85, Adjusted R-squared: 0.83, F-statistic: 39.0 (P value < 0.001)

mRS, modified Rankin Scale; PA periventricular anastomosis

### Unsupervised clustering of the cohort

To divide the cohort into clusters, principal component analysis was conducted. The dimension was first reduced to two (Fig. 2e) and then to three (Fig. 2f). Although cases with non-hemorrhagic onset PA aneurysms were indistinguishable from other cases, cases with hemorrhagic onset PA aneurysms were relatively distinct.

## Discussion

Our findings elucidated the frequency (2.7%) and associated factors of PA aneurysm (mRS score and PA score), distinguished hemorrhagic-onset from non-hemorrhagic-onset aneurysms, and demonstrated the aggressive clinical course of hemorrhagic-onset aneurysms and their risk for future hemorrhagic events.

Several studies have explored the relationship between intracranial aneurysms and MMD [17, 18, 24–27]. Among patients with MMD, the prevalence of intracranial aneurysms, classified as major artery and non-major artery aneurysms, ranges from 3.4 to 24.7% [18, 26, 27]. However, existing statistics often include PA aneurysms and aneurysms occurring within the circle of Willis. For PA aneurysms alone, the reported frequency ranges from 2.3 to 24.7% [18, 19], which is consistent with the 2.7% prevalence in our study cohort.

The risk of hemorrhage in PA aneurysms has been documented by several groups [17, 18, 25, 28]. One study used logistic regression analysis to show the association between PA aneurysm and hemorrhagic presentation [28]. Another study identified PA aneurysm as a significant risk factor for future hemorrhagic strokes based on multivariate Cox hazard regression analysis [25]. These results are similar to our study findings, which have identified PA aneurysm as a significant risk factor for future hemorrhagic strokes based on multivariate analysis. While studies have demonstrated the association between older age and PA aneurysm formation [18, 29], which was also revealed in our machine learning analysis, no such association was found in the multivariate analysis. However, higher PA scores and higher initial mRS scores were found to be significantly associated with PA aneurysm formation. Although higher initial mRS scores may be influenced by confounding factors, such as hemorrhagic presentation or future hemorrhagic events, the association with higher PA scores shows greater potential. Several authors have proposed that hemodynamic stress and vascular abnormalities contribute to the formation of PA aneurysms [15, 25, 28, 30]. However, none of these reports have demonstrated this association through statistical or mathematical analysis. Our study was able to demonstrate these associations by using the PA score as an indicator of vessel pathology. Indeed, the strong association between PA scores and PA aneurysms was confirmed using multivariate analysis and machine learning approaches. When considering the underlying pathophysiology of the association between higher PA scores and PA aneurysm formation, histopathological studies would aid in better understanding. In moyamoya disease, collateral vessels are characterized by proliferative changes in the intima, marked thinning of the media, and wavy contorted elastic lamina [31]. Additionally, a postmortem histological study revealed that dilated collateral moyamoya vessels or microaneurysms were composed of fragmented elastic lamina and attenuated media [32]. On the other hand, flow-driven wall shear stress is known to activate proinflammatory signaling in endothelial cells that recruit macrophages [33]. The macrophage infiltration is known to disrupt the internal elastic lamina and collagen matrix, which results in aneurysmal formation [33]. Hence, the vascular vulnerability and flow-driven wall-shear stress of the collateral vessels seem to predispose them to aneurysm formation and rupture. Higher PA scores imply that the pathological changes in the fragile collateral vessels are more prominent and that flow-driven wall shear stress is greater. Therefore, the association between higher PA scores and PA aneurysm formation may be explained by the more severe proliferative and degenerative changes in the collateral vessels and the greater wall shear stress imposed on them.

Regarding the terminology, it would be difficult to discern whether the aneurysm has truly ruptured, and using the term “rupture” may not be vigorous. However, as shown in Fig. 1, hemorrhagic-onset PA aneurysm-positive cases exhibited an irregular aneurysmal shape, and all cases possessed a bleb. Additionally, as shown in supplement 3, the intracerebral, intraventricular, and subarachnoid hemorrhage are distributed around the aneurysm. These were the rationale for us to use the term “rupture”.

Genotype analysis was additionally performed for certain patients in this study. The analysis showed that PA aneurysm-positive cases were more likely to carry the GG genotype (60%) compared to PA aneurysm-negative cases (31.4%). However, this difference did not reach statistical significance (*P* = 0.33), which may be partially due to the limited sample size. Given the implicated mechanism of *RNF213* in the pathophysiology of MMD, genetic analysis is a promising tool for predicting the clinical course and modifying patient management strategies [22, 34]. Ongoing research continues to accumulate evidence, highlighting the significance of genetic studies in this area. Further analysis is needed to elucidate the correlations among genotype, clinical features, and PA aneurysm development.

Our study revealed a vivid contrast in outcomes between hemorrhagic-onset and non-hemorrhagic-onset cases. At the last follow-up assessment, all hemorrhagic cases presented with an mRS score of 5. Conversely, non-hemorrhagic cases that did not experience hemorrhagic strokes or aneurysmal ruptures during the study period presented with an mRS score of 0–2. Additionally, a multivariate regression model applied to the hemorrhagic-onset group (33 cases) revealed that PA aneurysm was associated with a higher modified Rankin scale at the last follow-up. This distinction suggests that unruptured PA aneurysms pose a significantly lower risk compared to ruptured PA aneurysms. In addition, ruptured PA aneurysms are associated with worse outcomes, whereas unruptured PA aneurysms are not. These findings suggest that clinicians should address both cases in a different manner. Furthermore, unsupervised clustering analysis showed consistent findings in our study. Principal component analysis revealed that ruptured PA aneurysms were distinct from other MMD cases (Fig. 2d, e). Although unruptured and ruptured intracranial aneurysms within the circle of Willis are recognized as different disease entities [35], this distinction has yet to be accepted in the context of MMD. As such, we advocate the importance of differentiating unruptured from ruptured PA aneurysms when assessing patients with MMD.

Another aspect emphasized in our study is the risk of aneurysmal re-ruptures and rupture of other vessels in PA aneurysm-positive patients. This is exemplified by *Case 3*, who suffered from a re-rupture of the ChA aneurysm within a short interval of 1 month, whereas *Case 1* and *Case 2* experienced bleeding from other sites of the prominent RA at a relatively later interval of 39 months. Interestingly, similar cases of late-onset rebleeding in other sites have already been documented in a previous report [19]. This implies that, alongside hemostasis of the ruptured aneurysm, interventions targeting the fragile collateral vasculature within the PA network are equally crucial to prevent further clinical deterioration in these cases. However, some cases do not experience rebleeding even when observational strategies are opted for [15]. A few aneurysms may even regress spontaneously [36]. There is insufficient data regarding the differences between cases that experience re-rupture and cases that regress spontaneously. Further accumulation of cases, especially a larger dataset collected prospectively, would provide additional insights into this issue. Extensive analysis may enable the differentiation of high-risk groups and low-risk groups of re-rupture and would enable clinicians to decide whether early interventions or observations are desirable depending on each patient’s clinical status.

Taken collectively, our findings showed that PA aneurysms in MMD demonstrated significant prognoses and clinical courses depending on the presence of hemorrhage. Neurosurgeons should recognize the hemorrhagic risk of ruptured PA aneurysms, its association with further PA development, and the unfavorable prognosis of such cases. On the other hand, unruptured aneurysms present with a more favorable and benign clinical course, which should equally be recognized as well. Although our study population was limited, revascularization by trans-arterial embolization prevented further rupture in *Case 3* and *Case 4*, which is consistent with a previous report [15]. Therefore, clinicians should recognize the clinical courses of hemorrhagic and non-hemorrhagic cases, wherein interventions should be considered in hemorrhagic-onset PA aneurysms.

### Limitations

Despite the insights offered in this study, certain limitations should be acknowledged. First, given the single-center retrospective nature of this study, selection bias and limited sample size were inevitable. We were unable to generate sufficient validation data to rule out selection bias in the machine learning approaches, which we attempted to mitigate by randomly dividing our available data. Second, though we aimed to analyze the *RNF213* p.Arg4810Lys, c.14429G > A rs112735431 in all cases, only 164 cases could undergo the genetic analysis. Therefore, we were not able to include the genetic information in the multivariate analysis. Additionally, cases that did not undergo genotyping were classified as *RNF213* GA genotype negative and *RNF213* GG genotype negative in the machine learning approaches. Third, we focused on PA aneurysms in this study and did not examine the clinical features and significance of major artery aneurysms in MMD cases. Comparing PA aneurysms with major artery aneurysms would possess clinical significance and would provide additional insights. Lastly, while our study elucidated the clinical significance of PA aneurysms, we were unable to investigate the optimal management or treatment strategies of these cases. We eliminated the impact of surgery by excluding data at the point of surgery in order to simplify the analysis. Studies focusing on those therapeutic aspects of PA aneurysms would be essential for further analysis.

## Conclusions

In conclusion, PA aneurysms occurred in 2.7% of patients with MMD. PA aneurysms were associated with higher PA scores and suggested a higher risk of future hemorrhagic strokes. Hemorrhagic-onset PA aneurysms were associated with worse prognosis and were distinct from other MMD cases, whereas non-hemorrhagic-onset cases were relatively benign and indistinguishable. Clinicians should recognize that aneurysmal rupture of the periventricular anastomosis is associated with further disease progression and poor prognosis, requiring immediate and appropriate interventions.

## Electronic supplementary material

Below is the link to the electronic supplementary material.

Supplementary Material 1

## Data Availability

The datasets generated and analyzed during the current study are available from the corresponding author on reasonable request.

## Abbreviations

AChA: Anterior choroidal artery
AIC: Akaike information criterion
AUC: Area under the curve
CI: Confidence interval
ChA: Choroidal artery
CT: Computed tomography
DSA: Digital subtraction angiography
EDAS: Encephalo-duro-arterio-synangiosis
EFS: Event-free survival
EMS: Encephalo-myo-synangiosis
ICA: Internal carotid artery
LSA: Lenticulostriate artery
JAM trial: Japan Adult Moyamoya trial
MMD: Moyamoya disease
MR: Magnetic resonance
mRS: Modified Rankin scale
PA: Periventricular anastomosis
PCA: Posterior cerebral artery
PChA: Posterior choroidal artery
ROC: Receiver operating characteristic
SVM: Support vector machine
THA: Thalamic artery
