# Supplementary material for: Aneurysmal formation of periventricular anastomosis is associated with collateral development of Moyamoya disease and its rupture portends poor prognosis: detailed analysis by multivariate statistical and machine learning approaches
Source: Neurosurg Rev. 2024 Nov 19;47(1):856. doi: 10.1007/s10143-024-03097-2 (PMC11573815; doi:10.1007/s10143-024-03097-2)
Supplement: Supplementary file 1 — Supplementary Material 1 [file 10143_2024_3097_MOESM1_ESM.pdf]

	LSA	THA	AChA	PChA
Grade 0	No dilation and extension	No dilation and extension	No dilation and extension	No dilation and extension
Grade 1	Dilation or extension below the level of the pericallosal artery	Dilation or extension below the level of the MPChA	Dilation or extension below the level of the lateral ventricle	Dilation or extension below the level of the pericallosal artery (MPCHA) and the lateral ventricle (LPChA)
Grade 2	Dilation and extension beyond the level of the pericallosal artery	Dilation and extension beyond the level of the MPChA	Dilation and extension beyond the level of the lateral ventricle	Dilation and extension to the pericallosal artery (MPChA) and beyond lateral ventricle (LPChA)

***PA score was calculated as the sum of the individual grades of each collateral pathway (LSA, THA, and ChA).**
****ChA collateral grading was defined as the combined scores of AChA and PChA collaterals, taking the higher value for each.**

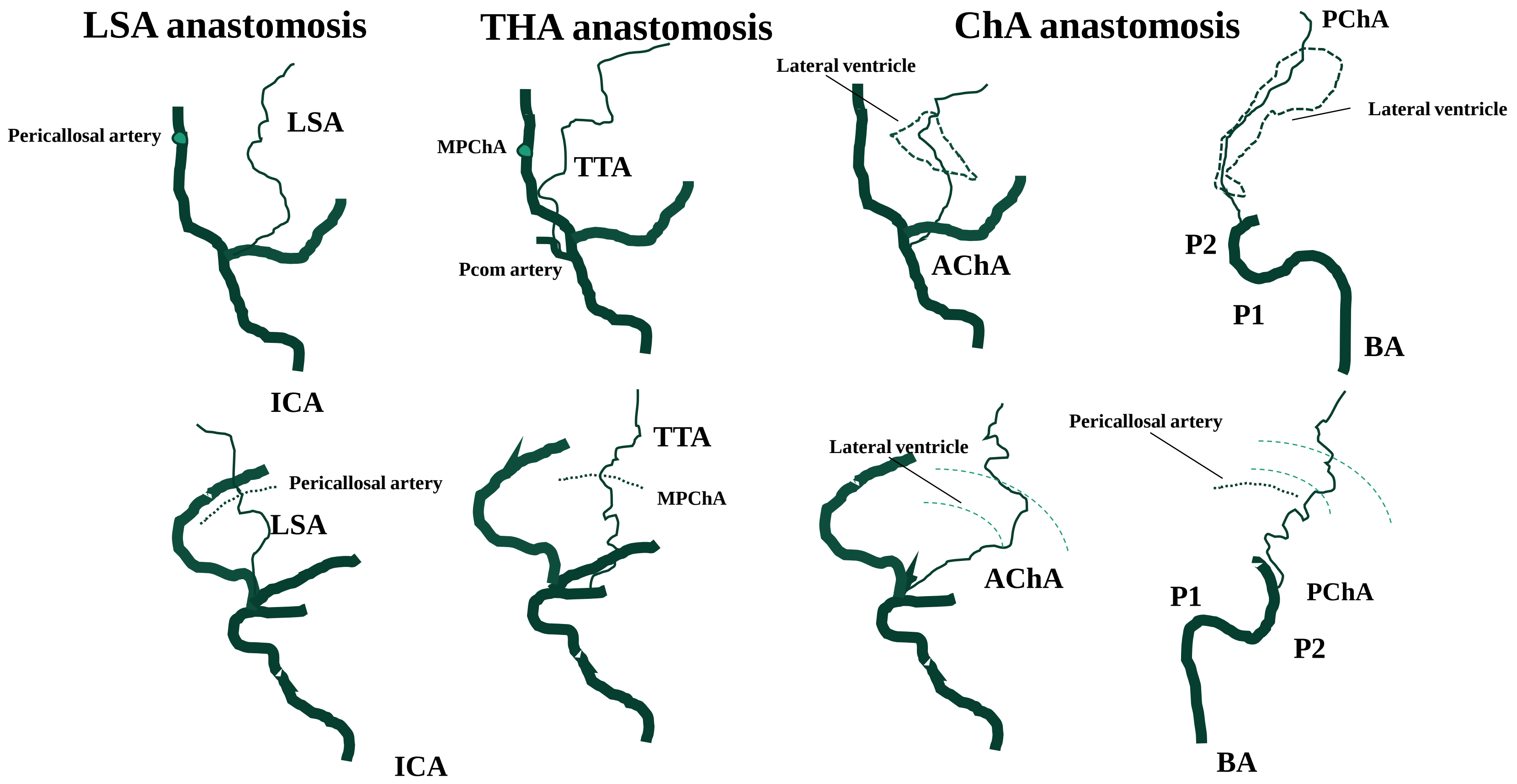

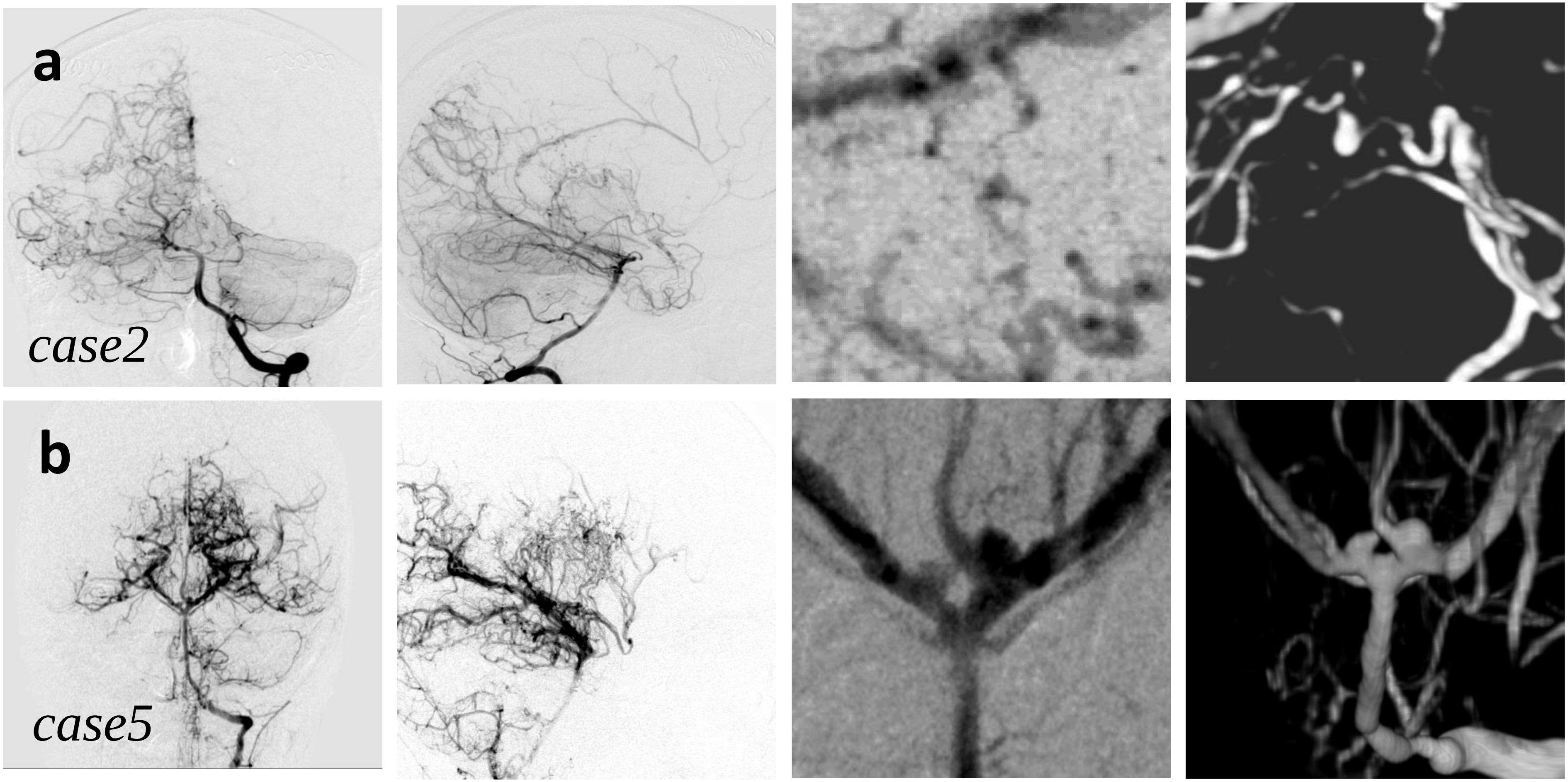
a
case2
b
case5

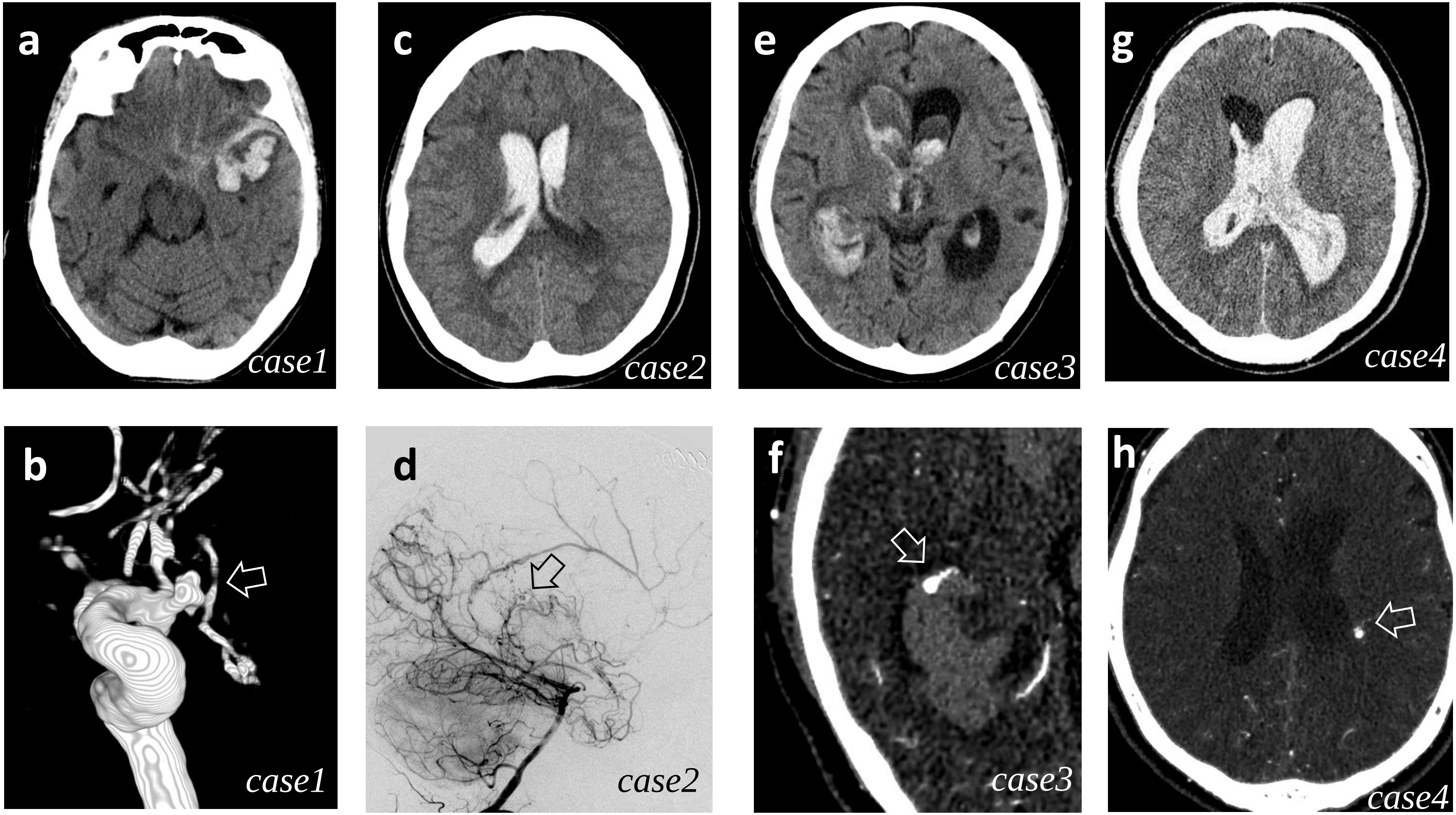
a
case1
c
case2
e
case3
g
case4
b
case1
d
case2
f
case3
h
case4

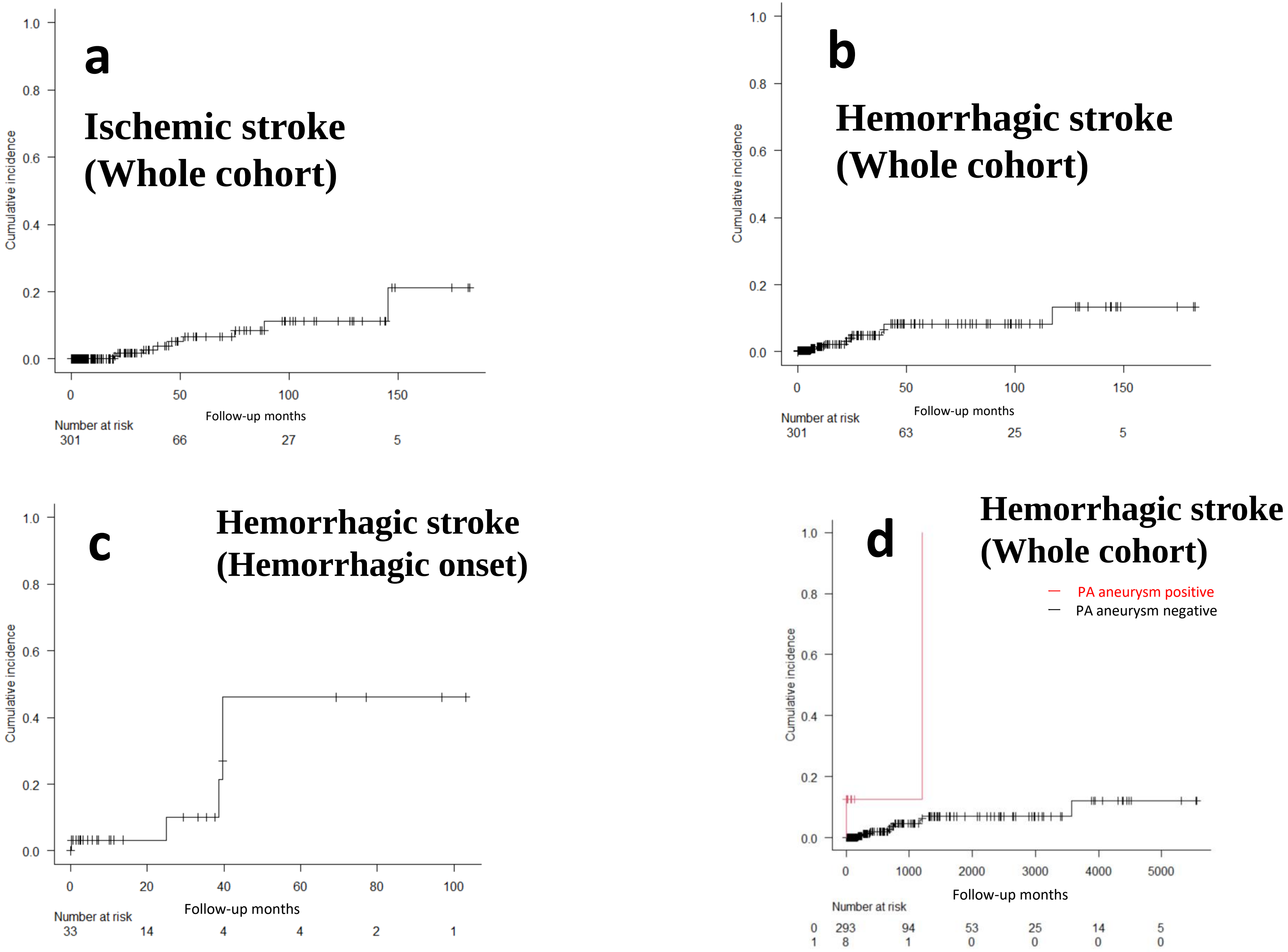
a
Ischemic stroke
(Whole cohort)
Cumulative incidence
1.0
0.8
0.6
0.4
0.2
0.0
0
50
100
150
Follow-up months
Number at risk
301
66
27
5
b
Hemorrhagic stroke
(Whole cohort)
Cumulative incidence
1.0
0.8
0.6
0.4
0.2
0.0
0
50
100
150
Follow-up months
Number at risk
301
63
25
5
c
Hemorrhagic stroke
(Hemorrhagic onset)
Cumulative incidence
1.0
0.8
0.6
0.4
0.2
0.0
0
20
40
60
80
100
Follow-up months
Number at risk
33
14
4
4
2
1
d
Hemorrhagic stroke
(Whole cohort)
PA aneurysm positive
PA aneurysm negative
Cumulative incidence
1.0
0.8
0.6
0.4
0.2
0.0
0
1000
2000
3000
4000
5000
Follow-up months
Number at risk
0 293 94 53 25 14 5
1 8 1 0 0 0 0
